# Cold Plasma Preparation of Pd/Graphene Catalyst for Reduction of p-Nitrophenol

**DOI:** 10.3390/nano11051341

**Published:** 2021-05-20

**Authors:** Qian Zhao, Decai Bu, Zhihui Li, Xiuling Zhang, Lanbo Di

**Affiliations:** College of Physical Science and Technology, Dalian University, Dalian 116622, China; 15732702250@163.com (Q.Z.); caizi1108@163.com (D.B.); lee6062@163.com (Z.L.); xiulz@sina.com (X.Z.)

**Keywords:** cold plasma, Pd, graphene, reduction of 4-NP, catalytic hydrogenation

## Abstract

Supported metal nanoparticles with small size and high dispersion can improve the performance of heterogeneous catalysts. To prepare graphene-supported Pd catalysts, graphene and PdCl_2_ were used as support and Pd precursors, respectively. Pd/G-P and Pd/G-H catalysts were prepared by cold plasma and conventional thermal reduction, respectively, for the catalytic reduction of p-nitrophenol (4-NP). The reaction followed quasi-first-order kinetics, and the apparent rate constant of Pd/G-P and Pd/G-H was 0.0111 and 0.0042 s^−1^, respectively. The graphene support was exfoliated by thermal reduction and cold plasma, which benefits the 4-NP adsorption. Pd/G-P presented a higher performance because cold plasma promoted the migration of Pd species to the support outer surface. The Pd/C atomic ratio for Pd/G-P and Pd/G-H was 0.014 and 0.010, respectively. In addition, the Pd nanoparticles in Pd/G-P were smaller than those in Pd/G-H, which was beneficial for the catalytic reduction. The Pd/G-P sample presented abundant oxygen-containing functional groups, which anchored the metal nanoparticles and enhanced the metal-support interaction. This was further confirmed by the shift in the binding energy to a high value for Pd3d in Pd/G-P. The cold plasma method operated under atmospheric pressure is effective for the preparation of Pd/G catalysts with enhanced catalytic activity for 4-NP reduction.

## 1. Introduction

In recent decades, environmental issues have become increasingly important worldwide, and the mitigation of water resource pollution has become a top priority owing to the large volume, long treatment cycles, and complicated processes. Among the several pollutants of interest, 4-nitrophenol (4-NP) is present in industrial wastewater and has a strong toxic effect. It can cause systemic poisoning when absorbed through the skin or respiratory tract. It has also been listed as a priority pollutant by the US Environmental Protection Agency [1]. The catalytic reduction of 4-NP into 4-aminophenol (4-AP) by supported metal catalysts, which has low toxicity, is an effective method that can be applied for the safe discharge of industrial wastewater [2,3,4]. Several methods, including photoreduction, impregnation, and chemical reduction, have been used to prepare supported metal catalysts to minimize the content of 4-NP in industrial wastewater [5,6]. The photoreduction method has high catalytic performance and can provide small particle size metal nanoparticles. However, it has a high cost and long reaction time, thus it is difficult to obtain a high-load metal catalyst. The impregnation method is simple and inexpensive, but the obtained metal nanoparticles are large, unevenly distributed, and have low catalytic performance. The chemical reduction method has good catalytic performance, but the prepared metal nanoparticles agglomerate easily, and the preparation process requires the use of toxic reducing agents and several many additives, which is unfavorable for the environment. Therefore, a fast, simple, and environmentally friendly method should be developed for the preparation of high-performance supported metal catalysts. In recent years, the use of atmospheric pressure cold plasma-assisted impregnation has attracted increasing attention for this purpose [7].

Atmospheric pressure cold plasma is a green and efficient method for preparing supported metal catalysts, and it normally uses hydrogen active species generated from hydrogen-containing gas to reduce metal ions. Plasma treatment can generate a variety of reactive species, such as high-energy electrons and hydrogen radicals, which can effectively reduce graphite oxide to reduced graphene oxide and K_2_PtCl_4_ to PtNP [8]. In addition, the plasma reduction of graphene-based materials is accompanied by multiple surface modification processes such as hydrogenation, etching, and exfoliation, which increase the catalyst loading and dispersibility while improving its activity. Liu et al. [9] obtained in situ exfoliated edge-oxygen-enriched functionalized graphene using Ar plasma to treat the surface of carbon fibers. Cardinali et al. [10] used argon plasma to process multilayer graphite oxide (GO) sheets at room temperature, and their results showed that the number of rGO layers was significantly reduced; and the specific surface area and light transmittance significantly increased, which demonstrated the peeling effect of the plasma on the multilayer graphene. Yang et al. [11] modified GO using both reducing and depositional CH_4_/Ar plasma, and found that when GO was reduced, the defect rate was also reduced, and the carbon-to-oxygen ratio significantly increased. This occurred because the active particles (such as CH_x_, C, and H) generated by the decomposition of CH_4_ molecules in the plasma are reductive; as such, they react with oxygen-containing radical groups and remove them, whereas carbon-containing particles are easily deposited because they are non-volatile. These active particles easily react with the defect active vacancies or edges of GO and are then deposited there, which benefit the repairing of acupoint defects or epitaxial growth. Di [12] prepared Pd/C nanocatalysts using dielectric barrier discharge (DBD) cold plasma and obtained high catalytic activity and stability in the dehydrogenation of formic acid. Ren [13] synthesized graphene and Pd nanoparticle hybrids via the plasma method and obtained excellent catalytic activity in the Suzuki coupling reaction. Dameron et al. [14] used O_2_/Ar plasma generated by an inductively coupled plasma generator to pretreat carbon nanotubes and functionalize them to support Pt nanoparticles. The experimental results showed that the obtained Pt-based catalyst exhibited good electrochemical activity. Xu et al. [15] used room-temperature hydrogen plasma to reduce a mixture of GO and palladium chloride (PdCl_2_). During the impregnation process, because GO contains a large amount of oxygen-containing functional groups (e.g., –COOH, –CO, and –OH), the Pd^2+^ in the solution was physically adsorbed, and electrostatic or charge transfer interactions occurred on the GO surface to form a Pd^2+^/GO complex. The Pd^2+^/GO complex was treated with hydrogen plasma to simultaneously reduce Pd^2+^ and GO. The final result was a composite material with 2 nm Pd nanoparticles supported on the graphene surface. The Pd nanoparticles were well dispersed, and the carbonyl sulfide hydrogenation reaction exhibited a high catalytic efficiency. Therefore, graphene and GO have great potential as supports due to their large specific surface area and unique three-dimensional fold structure.

Graphene and GO are two new types of carbon material, with unique structure and properties such as large surface area, excellent thermal and chemical stability, high electrical conductivity, and large number of oxygen-containing functional groups. In addition, their unique three-dimensional structure increases its potential as a nanoparticles support. They are considered ideal materials in optoelectronic devices [16], supercapacitors [17], hydrogen storage devices [18,19], sensors [20], and catalysts [21]. Studies have shown that there is a synergistic catalytic effect between graphene/GO and metal nanoparticles [22]. This can effectively improve the catalytic activity and stability. Wu et al. [23] reported a two-step method for the preparation of Au-Ag alloy NP-decorated GO nanosheets. The preparation process required the addition of a NaOH solution to immobilize metal nanoparticles. The catalyst exhibited excellent catalytic activity for the reduction of 4-NP by NaBH_4_ at room temperature. The reaction time was 5 min, and the first-order rate constant k was 0.761 min^−1^. Revathy et al. [24] used the NaBH_4_ reduction method to synthesize a graphene-supported palladium-nickel alloy catalyst to reduce 4-NP to the environmentally friendly 4-AP. The reaction time was 12 min, and the reaction rate constant was k was 0.16 min^−1^. Liu et al. [25] used nitrogen-doped GO as a support to prepare a Ni_0.5_Pd_0.5_/NrGO catalyst by chemical reduction. Ni_0.5_Pd_0.5_/NrGO catalyzed the reduction of 4-NP to 4-AP, and the reaction rate constant k was 0.0170 s^−1^. In a recent study by Minati et al. [26], graphene xerogels with Pd were prepared by a hydrothermal assembly method and used as composite catalysts for the catalytic reduction of rhodamine 6G, which has a highly porous and strong structure. The composite catalyst was added to a mixed solution of rhodamine 6G solution and NaBH_4_. The solution became colorless in approximately 3 min, and the reaction rate constant k was 0.01935 s^−1^. Although some chemical reducing agents (such as hydrazine and NaBH_4_) can reduce metal ions and/or GO, their toxicity and structural damage to the graphene/GO supports are challenges that still need to be overcome [27,28,29]. Moreover, among the metal catalysts, graphene-based carbon materials supported Pd is prevailing due to their high performance. This is because Pd has a large hydrogen storage capacity, and the migration of H atoms from Pd to graphene-based carbon materials is favorable due to the strong metal-support interaction [2]. Therefore, designing a simple, efficient, and green method to prepare high performance graphene-based carbon materials supported Pd catalysts for the reduction of 4-NP is needed.

In this study, graphene-supported Pd catalysts (Pd/G-P and Pd/G-H) for the reduction of 4-NP were prepared by cold plasma and conventional thermal reduction, respectively. The reaction followed a quasi-first-order reaction, and the apparent rate constant for Pd/G-P was 0.0111 s^−1^, which was 2.64 times that of Pd/G-H (0.0042 s^−1^). Various methods were used to determine the structure–performance relationship of the catalysts. The high performance of Pd/G-P was ascribed to the high atomic ratios of Pd/C and O/C, as well as the enhanced metal-support interaction.

## 2. Experimental

### 2.1. Materials

All reagents purchased were of analytical grade and were used without further purification. Palladium chloride (PdCl_2_) was purchased from Tianjin Kermel Chemical Reagent Co., Ltd (Tianjin, China). Multilayer graphene (6–10 layers) was purchased from Suzhou Carbon Graphene Company (Suzhou, China), and 4-nitrophenol (4-NP) was purchased from Shanghai Aladdin Chemical Reagent Co., Ltd (Shanghai, China). NaBH_4_ was purchased from Tianjin Komiou Chemical Reagent Co., Ltd (Tianjin, China). Argon (>99.999%) and hydrogen (>99.999%) were provided by Zhonghao Bright Chemical Research and Design Institute Co., Ltd (Dalian, China).

### 2.2. Preparation of Palladium–Graphene Catalyst

#### 2.2.1. Catalyst Precursor

The Pd/G catalyst precursor was prepared with a Pd loading of 5 wt% by an excessive impregnation method. A certain mass of palladium chloride was weighed and dissolved in dilute hydrochloric acid to prepare a H_2_PdCl_4_ solution. Subsequently, 1 g of graphene was weighed into a beaker, to which a certain volume of distilled water was added and sonicated for 1 h. After that, an appropriate amount of H_2_PdCl_4_ solution was added according to the respective calculation, and the mixture was evenly stirred using a magnetic stirrer for 6 h. The sample was dried in a blast-drying oven at 120 °C for 2 h, and stirred evenly, which allowed the palladium ions to be more uniformly dispersed on the graphene surface to prepare the precursor of the Pd/G sample, which was labeled Pd/G-As.

#### 2.2.2. Pd/Graphene Catalyst

The dried Pd/G-As powder was reduced by hydrogen thermal reduction (Pd/G-H) and hydrogen cold plasma (Pd/G-P), and the obtained samples were denoted Pd/G-H and Pd/G-P, respectively.

For the preparation of Pd/G-P, 0.1 g of Pd/G-As was prepared using a coaxial dielectric barrier discharge (DBD) reactor, which was composed of a quartz tube (I.D. 7 mm, O.D. 10 mm), high-voltage electrode, and ground electrode. The high-voltage electrode (diameter: 2 mm), made of copper, was placed in the middle of the quartz tube. The quartz tube was wrapped by the ground electrode (length: 20 mm), which was made of an aluminum foil. The discharge gap and the discharge volume were 2.5 mm and 706.5 mm^3^. A self-made cold plasma power supply capable of generating a sinusoidal high voltage was used to generate plasma and discharge [30]. The discharge voltage, frequency, and time were 12.8 kV, 11.75 kHz, and 20 min. In the hydrogen plasma, a mix of H_2_ and Ar (H_2_:Ar = 1:1) was used as the working gas, and the prepared catalyst was labeled Pd/G-P.

To prepare the Pd/G-H, 0.1 g of Pd/G-As was placed in the middle of a quartz glass tube, which was placed in the middle of a tubular resistance furnace. The pipeline was connected, and the air tightness was checked. A hydrogen atmosphere was used for the reduction, at a gas flow rate of 100 mL∙min^−1^ for 2 h and temperature of 300 °C [31]. The sample was then naturally cooled to room temperature to obtain the Pd/G-H catalyst.

### 2.3. Catalyst Characterization

Powder X-ray diffraction (XRD) was used (Dandong Haoyuan DX-2700, Dandong, China) at a 2θ range of 5°–85° at the scanning rate of 4°·min^−1^, 40 kV, and 30 mA, using Cu-Kα radiation (λ = 0.154 nm). X-ray photoelectric spectroscopy (XPS), using monochromatic AlKα X-ray (1486.6 eV) source, was adopted to investigate the surface chemical composition of the samples on ESCALAN250 X-ray photoelectron spectrometer (Thermo VG, Waltham, MA, USA). The samples were characterized without any further pretreatment, and they were transferred in air. The binding energy of all elements was calibrated according to the C1s orbital binding energy at 284.6 eV. Transmission electron microscopy (TEM) images were captured using an HT7700 transmission electron microscope (Hitachi, Tokyo, Japan) at an acceleration voltage of 120 kV. The average size and size distribution of palladium nanoparticles were calculated by selecting no less than 100 palladium nanoparticles from the TEM image. The Fourier transform (FT)-Raman spectra of Pd/graphene were recorded by HORIBA JY LabRAM HR Evolution (HORIBA Scientific, Paris, France) (peak position calibrated at 520.7 with a silicon wafer). The surface morphology of the samples was obtained using a scanning electron microscope (SEM, Zeiss Sigma500, Jena, Germany). Spectrophotometric analysis was performed using a UV-3900 spectrophotometer (Hitach, Tokyo, Japan).

### 2.4. Activity Test

The Pd/G catalytic hydrogenation of 4-NP was conducted at room temperature. Typically, to ensure the accuracy of the test, 4 mg of Pd/G catalyst was dispersed in 40 mL of 4-NP (0.3 mM) aqueous solution and magnetically stirred for 5 min. Subsequently, 3 mL of a NaBH_4_ (0.1 M) solution was injected into the above mixture under continuous stirring. To evaluate the progress of the reaction, 3 mL of the solution was removed from the reaction mixture and placed in a quartz cuvette (1 cm). The catalytic process was monitored using an ultraviolet-visible spectrophotometer in the range of 250–500 nm at constant intervals.

## 3. Results and Discussion

**Figure 1:**
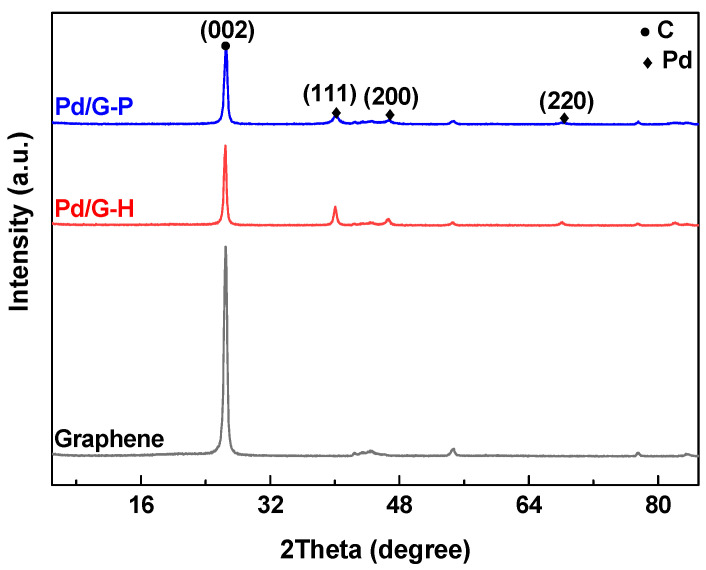
X-ray diffraction patterns of Pd/G-H, Pd/G-P, and graphene.

Figure 1 shows the XRD patterns of the Pd/G-H, Pd/G-P, and graphene support. Graphene, Pd/G-H, and Pd/G-P all presented a sharp characteristic diffraction peak at 26.5°, which corresponds to the (002) plane of multilayer graphene (PDF#97-002-9123). This diffraction peak was generated by the stacking of graphene sheets. Pd/G-H and Pd/G-P exhibited obvious characteristic diffraction peaks at 2θ = 40.1°, 46.7°, and 68.1° which correspond to the (111), (200), and (220) planes (PDF#97-005-2251) of metal Pd, respectively, with a face-centered cubic crystal structure. This demonstrates that both methods can reduce palladium ions to metallic palladium. Other characteristic Pd diffraction peaks were not observed. Compared with the graphene support, the intensity of the graphene diffraction peak in the catalysts after the loading of Pd species was significantly reduced. This was attributed to the covering effect of the Pd component and the effect of graphene exfoliation. As a result of the graphene exfoliation, it presented an ordered layer structure, which weakened the π-π stacking between the graphene layers [32,33]. The results indicate that the preparation of catalysts by different methods and the loading of metal palladium nanoparticles on graphene did not damage the basic graphene structure.

**Figure 2:**
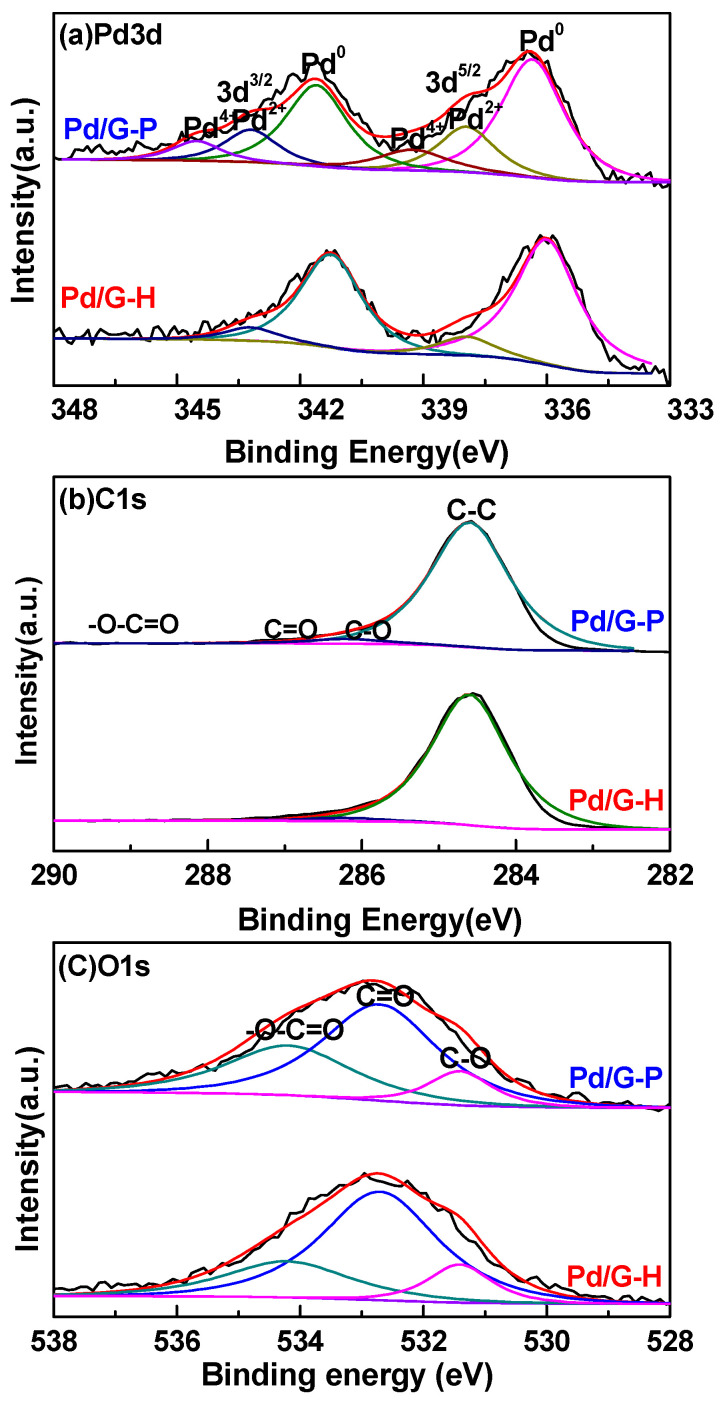
X-ray photoelectric spectroscopy spectra of (**a**) Pd3d, (**b**) C1s, and (**c**) O1s in Pd/G-H and Pd/G-P (black line: original data, red line: simulated data).

XPS was used to test the Pd/G-H and Pd/G-P catalysts and to analyze their elemental composition. Figure 2a shows the energy spectrum of Pd3d. After peak fitting, the Pd3d5/2 of Pd/G-H presented two characteristic peaks with binding energies of 336, and 337.25 eV which corresponded to the valence states of Pd^0^ and Pd^II^, respectively [12,34,35]. The Pd3d5/2 of Pd/G-P can be fitted with three peaks, corresponding to the valence states of Pd^0^, Pd^II^, and Pd^IV^. Interestingly, the binding energies shifted to the direction of high binding energy (0.35 eV), mainly because of the strong interaction between the Pd nanoparticles and the graphene support [36]. The relative percentage of Pd on the surface of the Pd/G catalyst was calculated based on the peak areas obtained by fitting the Pd/G-H and Pd/G-P samples. As shown in Table 1, the Pd^0^ and Pd^II^ contents of Pd/G-H were 89.03% and 10.97%, while the Pd^0^, Pd^II^, and Pd^IV^ contents of Pd/G-P were 67.45%, 21.02%, and 11.53%, respectively. Because the graphene surface contains a large number of oxygen-containing functional groups, neither hydrogen thermal reduction nor cold plasma treatment methods can completely reduce the palladium ions. This phenomenon has also been observed in previous studies [37,38]. In addition, the valence states of Pd^II^ and Pd^IV^ in Pd/G-P than that in Pd/G-H may be ascribed to the small and broader size distribution of Pd nanoparticles, which were active in air. This will be investigated in the future.

The C1s peaks of Pd/G-H and Pd/G-P in Figure 2b were mainly overlapping and fitted to four peaks. The major peak at 284.6 eV was attributed to graphitic carbon, which indicates that most C atoms were still in the honeycomb lattice of graphene, and graphene was not destroyed [25,39]. This is consistent with the XRD results. The binding energy peaks at 286.2, 287.8, and 289.3 eV were attributed to C–O, C=O, and –O–C=O functional groups, respectively. Figure 2c shows the peak fitting of the O1s peak. The O1s peaks of Pd/G-H and Pd/G-P were mainly overlapped and fitted with three peaks. The major peak with a binding energy of 532.7 eV was attributed to carbonyl or aldehyde groups [40], whereas those at 531.4 and 534.2 eV were attributed to hydroxy, ester, or carboxyl groups [40,41]. This is consistent with the peak splitting results for C1s.

According to the XPS spectra of Pd3d, C1s, and O1s, the atomic ratios of Pd/C and O/C were obtained for Pd/G-H and Pd/G-P. Table 1 shows that the Pd/C atomic ratios in Pd/G-H and Pd/G-P were 0.010 and 0.014, respectively. The high Pd/C atomic ratio in Pd/G-P occurred because cold plasma is a fast low-temperature process. In cold plasma, the electron mobility is significantly greater than that of the metal nanoparticles. A large number of electrons move toward the pores or inner surface of the support, which can promote the migration of negatively charged metal nanoparticles to the outer surface of the support [42]. In addition, the etching effect of cold plasma on graphene causes the graphene surface to be enriched with more active species of Pd nanoparticles. The O/C atomic ratio in Pd/G-H and Pd/G-P was 0.056 and 0.069, respectively. The O/C ratio of Pd/G-H was low because the conditions of hydrogen thermal reduction (300 °C and 2 h reaction time) led to the removal of some oxygen-containing functional groups. During the catalyst preparation by cold plasma, the reaction conditions were relatively mild, and only a small part of the oxygen was removed. In addition, hydrogen ions reacted with the oxygen on the graphene surface to form –OH, finally fixing on the catalyst surface. In addition, another likely reason for the high Pd/C atomic ratio of Pd/G-P was its abundance of oxygen-containing functional groups, which potentially anchored metal nanoparticles and strengthened the metal interaction between nanoparticles and graphene support.

**Figure 3:**
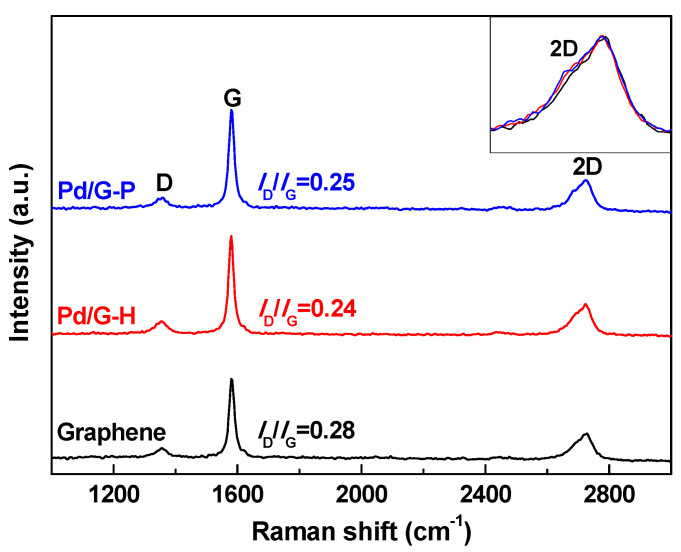
Fourier transform Raman spectra of graphene, Pd/G-H and Pd/G-P.

Figure 3 shows the FT-Raman spectra of Pd/G-H, Pd/G-P, and graphene support. The graphene support presented three peaks at 1359, 1589, and 2739 cm^−1^. The D peak was used to measure the degree of structural disorder. The G peak of the in-plane stretching vibration of sp^2^ hybridization of carbon atoms and the 2D peak reflecting the number of graphene layers [43]. *I*_D_/*I*_G_ were calculated by the integrated intensity ratio under the 1359 and 1589 cm^−1^ lines. The *I*_D_/*I*_G_ value is typically used to characterize the degree of defects in carbon material structures [44]. The *I*_D_/*I*_G_ values of graphene, Pd/G-H, and Pd/G-P were 0.28, 0.24, and 0.25, respectively. The defects in Pd/G-H and Pd/G-P were not significantly different. Compared with the graphene support, the defects were reduced to a certain extent in Pd/G-H and Pd/G-P. This occurred because of the loss of oxygen-containing functional groups on the graphene surface. The thermal reduction of hydrogen and the hydrogen cold plasma were both crucial for the graphene repair. The 2D peaks of Pd/G-H and Pd/G-P were not significantly different from those of graphene (inset of Figure 3). In addition, the 2D peak position of graphene was observed at 2727 cm^−1^, whereas those of Pd/G-H and Pd/G-P were 3 cm^−1^ to the left. Therefore, it can be concluded that Pd/G-H and Pd/G-P peeled off, which was consistent with the XRD results. Nevertheless, the number of graphene layers was not less than five [45].

**Figure 4:**
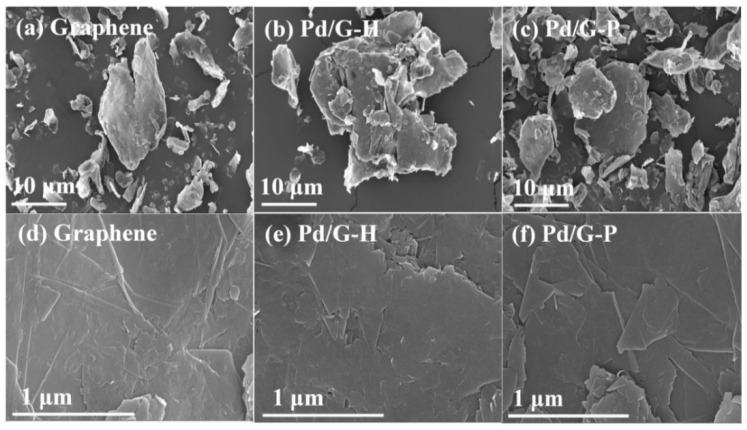
Scanning electron microscopy images of (**a**,**d**) graphene, (**b**,**e**) Pd/G-H, and (**c**,**f**) Pd/G-P.

Figure 4 shows SEM images of the graphene, Pd/G-H, and Pd/G-P catalysts. The graphene layering phenomenon was observed under low magnification (Figure 4a–c). Observations at higher multiples are shown in Figure 4d–f. The surface of the graphene sample was relatively complete and smooth because as the number of sheets increased, the graphene surface presented fewer wrinkles and tended to be flat, and the sheets were attracted to each other through van der Waals forces. Wrinkles were observed on the surface of Pd/G-H and Pd/G-P, and the graphene sheets appeared to be staggered and arranged together [46]. This was mainly caused by the graphene exfoliation, which was consistent with the XRD and Raman results. The exfoliation of Pd/G-H occurred because of the high temperature during the preparation process, which caused the oxygen-containing groups between the layers and the oxygen-containing groups at the edge of the sheet to form small molecules and escape. Under these circumstances, graphene sheets can overcome the van der Waals force for exfoliation. The peeling phenomenon of Pd/G-P occurred because cold plasma provides a large number of high-energy free electrons, which can bombard graphene in a hydrogen atmosphere to generate hydrogen molecules and free radicals and then remove the surface oxygen-containing functional groups. The generated water vapor and CO_2_ created an instantaneous high pressure, which caused the peeling effect. Figure 4e,f show that both hydrogen thermal reduction and cold plasma can cause a small amount of graphene exfoliation during the Pd/G preparation. However, neither of these two methods can exfoliate graphene to fewer than five layers (Figure 4b,c), which corresponds to the Raman results. In summary, the preparation of Pd/G catalysts through hydrogen thermal reduction or hydrogen cold plasma are both beneficial to the exfoliation of multilayer graphene.

**Figure 5:**
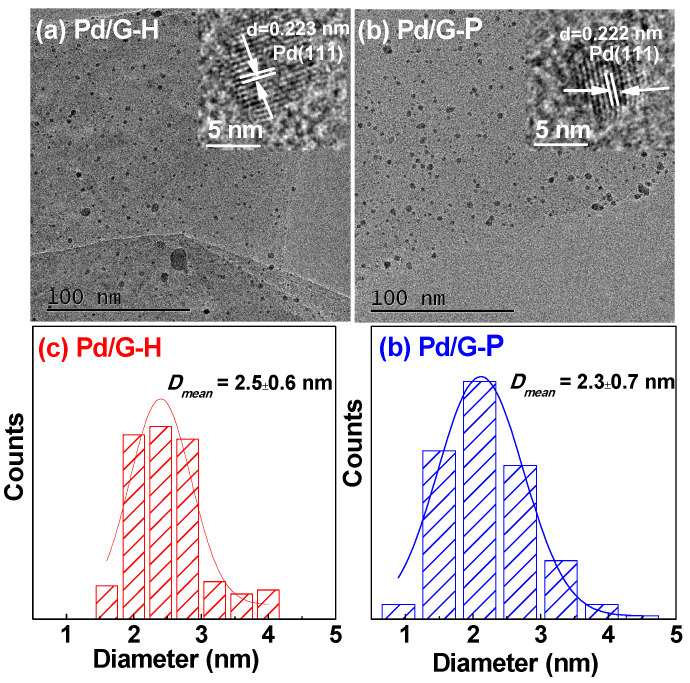
Transmission electron microscopy images of (**a**) Pd/G-H and (**b**) Pd/G-P, and (**c**,**d**) the corresponding particle size distribution histograms.

Figure 5 shows typical TEM images of Pd/G-H and Pd/G-P catalysts. The Pd nanoparticles were uniformly dispersed on the graphene surface in Pd/G-H and Pd/G-P (Figure 5a,b). Moreover, the HRTEM images of Pd/G-H and Pd/G-P show clear lattice fringes. The fringe spacing of 0.223 and 0.222 nm correspond to the (111) crystal lattice spacing of the face-centered cubic Pd structure. Based on the particle size distribution histograms of Pd/G-H and Pd/G-P catalysts (Figure 5c,d), the average particle size of Pd in Pd/G-H and Pd/G-P was calculated as 2.5 ± 0.6 and 2.3 ± 0.7 nm, respectively. These results demonstrate that small and highly dispersed Pd nanoparticles can be obtained by cold plasma. This occurred because the rapid and low-temperature process of cold plasma provided an adequate environment for rapid nucleation and crystal growth of metal nanoparticles [42]. In summary, compared with hydrogen thermal reduction, the cold plasma method can be applied at lower temperatures and with shorter reaction times, which is beneficial for the formation of smaller active Pd species.

**Figure 6:**
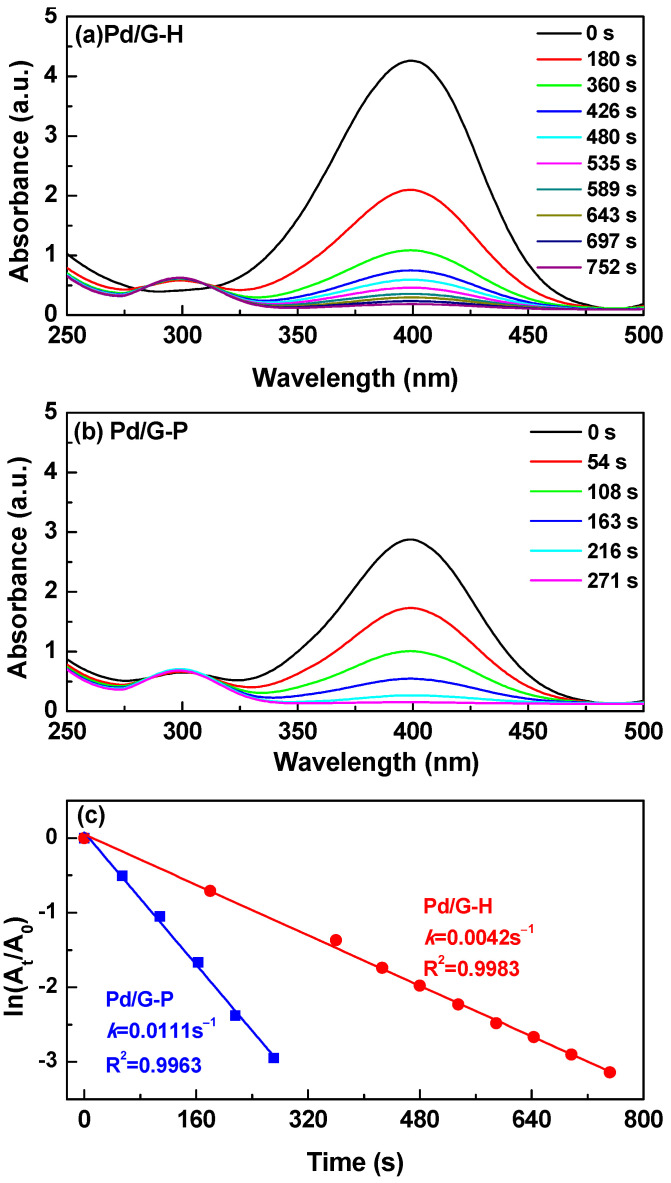
UV-Vis absorption spectra of (**a**) Pd/G-H and (**b**) Pd/G-P for the reduction of p-nitrophenol and (**c**) the corresponding graph of ln(*A*_t_/*A*_0_) vs. reaction time of 4-NP catalytic hydrogenation.

The catalytic activity of the prepared Pd/G catalyst was evaluated by the NaBH_4_ catalytic reduction of 4-NP to generate a 4-AP model system [47,48]. Figure 6a,b show the UV–Vis adsorption spectra of Pd/G-H and Pd/G-P catalysts for the reduction of 4-NP. As the catalytic reaction progressed, the absorption peak of 4-NP at 400 nm gradually weakened, and the absorption peak at 300 nm gradually increased. This new peak was attributed to the 4-AP. The adsorption peak of 4-NP disappeared, indicating that the hydrogenation reactions converted all 4-NP to 4-AP [49]. The time required for the complete conversion of 4-NP to 4-AP was 752 and 271 s for the Pd/G-H and Pd/G-P catalysts, respectively, which reflects the superior catalytic performance of Pd/G-P. Figure 6a,b also show that two isostatic points of the catalytic reaction appear at 280 and 314 nm, which indicates that when the Pd/G catalyst reduced 4-NP with NaBH_4_, no byproducts were formed. During the 4-NP reduction, the Pd nanoparticles dispersed on the graphene absorbed the hydrogen produced by the NaBH_4_ [24]. Simultaneously, 4-NP was adsorbed onto the catalyst surface, which increased the contact between the reactants and the catalyst and facilitated the rapid conversion of 4-NP to 4-AP [48].

To further analyze the kinetics of the 4-NP catalytic reduction, the relative peak intensity at 400 nm of the UV–Vis spectrum (Figure 6a,b) was measured, with an ln(*A*_t_/*A*_0_) plot of time t. The data fitting indicated that the R^2^ values of Pd/G-H and Pd/G-P were 0.9983 and 0.9963 (Figure 6c), respectively, which demonstrates their linear relationship and indicates that the reaction follows quasi-first-order kinetics. Because the initial NaBH_4_ concentration greatly exceeded that of 4-NP (by approximately 300 times), we assumed that the reduction rate was not related to NaBH_4_. Therefore, the hydrogenation reduction reaction of 4-NP follows the Langmuir–Hinshelwood model [50]. In fact, no catalytic activity was detected without any catalyst. The quasi-first-order law kinetic equation of the reaction can be expressed as $-\mathrm{In}\frac{A_{t}}{A_{0}}=kt$, where *A*_t_ and *A*_0_ represent the absorbance at a certain moment and at the initial stage, respectively, t is the time, and k is the kinetic rate constant [51]. The rate constant (*k*) was determined by the slope of the linear ln(*A*_t_/*A*_0_) with respect to the graph, and it represents the reaction speed. According to the slope, the catalytic reaction rate constants of Pd/G-H and Pd/G-P were 0.0042 and 0.0111 s^−1^, respectively. Relevant literature results concerning reduction of 4-NP by different catalysts have been illustrated in Table 2, from which we can see that the plasma prepared Pd/G-P has a much higher k value. The catalytic reaction rate constant of Pd/G-P was 2.64 higher than that of Pd/G-H. These results show that the catalytic activity of Pd/G-P was significantly higher than that of Pd/G-H because the former has abundant oxygen-containing functional groups, which can anchor the metal nanoparticles and enhance the metal-support interaction. Pd nanoparticles were small in size and highly dispersed on the graphene surface after cold plasma treatment. After the plasma treatment, the Pd nanoparticles shifted to a high binding energy, which enhanced the electronic interaction between the Pd nanoparticles and the graphene support. In addition, the weak mutual attraction of the π bonds between graphene and 4-NP also promoted the 4-NP adsorption [2].

## 4. Conclusions

In this work, Pd/G-P and Pd/G-H catalysts for the catalytic reduction of 4-NP were prepared by cold plasma and conventional thermal reduction methods, respectively. The reaction followed quasi-first-order kinetics, and the apparent rate constant for Pd/G-P was 0.0111 s^−1^, which was 2.64 times that of Pd/G-H (0.0042 s^−1^). Cold plasma applied under atmospheric pressure was effective for the preparation of Pd/G catalysts with enhanced catalytic activity for 4-NP reduction. Various methods were used to determine the structure–performance relationship of the catalysts. The high performance of Pd/G-P was ascribed to the high atomic ratios of Pd/C and O/C, as well as the enhanced metal-support interaction. Overall, plasma is a safe, efficient, and green method for the preparation of graphene-supported Pd catalysts for the catalytic reduction of 4-NP.

## Figures and Tables

**Table 1:** Pd composition and atomic ratio of Pd/C and O/C in Pd/G-H and Pd/G-P catalysts.

Sample	Pd Composition (%)			Pd/C Atomic Ratio	O/C Atomic Ratio
	Pd^0^	Pd^II^	Pd^IV^		
Pd/G-H	89.03	10.97	-	0.010	0.056
Pd/G-P	67.45	21.02	11.53	0.014	0.063

**Table 2:** Relevant literature results for reduction of 4-NP by different catalysts.

Catalyst	Concentration of 4-NP (mM)	Concentration of NaBH_4_ (mM)	Time (min)	*k* (min^−1^)	Ref.
Pd/G-P (5 w%)	0.3	100	4.52	0.660	This work
Pd/G-H (5 w%)	0.3	100	12.54	0.252	This work
Pd/rGO (1 w%)	10	10	180	0.0051	[2]
Pd/C (5 w%)	10	10	210	0.0064	[2]
Pd/PNGO (1 w%)	10	10	20	0.1676	[2]
Au-Ag/GO(ΙΙ)	1.54	880	5	0.761	[23]
Pd-Ni/rGO (30 w%)	0.5	30	12	0.16	[24]
Ni_0.5_Pd_0.5_/NrGO	0.05	100	2.67	1.02	[25]
